# High-flow nasal oxygenation reduces the risk of desaturation in adults receiving procedural sedation: a meta-analysis of randomized controlled trials

**DOI:** 10.1186/s13741-021-00212-5

**Published:** 2021-12-06

**Authors:** Hsin-Yi Liu, Ka-Wai Tam, El-Wui Loh, Wan-Chi Liu, Hsien-Cheng Kuo, Chun-Cheng Li, Yih-Giun Cherng, Jui-Tai Chen, Ying-Hsuan Tai

**Affiliations:** 1 Department of Anesthesiology, Shuang Ho Hospital, Taipei Medical University, No. 291, Zhongzheng Rd., Zhonghe District, New Taipei City, 23561 Taiwan; 2 Department of Anesthesiology, School of Medicine, College of Medicine, Taipei Medical University, Taipei, Taiwan; 3 Division of General Surgery, Department of Surgery, Shuang Ho Hospital, Taipei Medical University, New Taipei City, Taiwan; 4 Department of Surgery, School of Medicine, College of Medicine, Taipei Medical University, Taipei, Taiwan; 5 Cochrane Taiwan, Taipei Medical University, Taipei, Taiwan; 6 Department of Dentistry, Shuang Ho Hospital, Taipei Medical University, New Taipei City, Taiwan; 7 Graduate Institute of Clinical Medicine, College of Medicine, Taipei Medical University, Taipei, Taiwan

**Keywords:** Conscious sedation, High-flow nasal cannula, Hypoxia, Oxygen therapy, Patient satisfaction

## Abstract

**Background:**

Procedural sedation reduces patients’ discomfort and anxiety, facilitating performance of the examination and intervention. However, it may also cause adverse events, including airway obstruction and hypoxia. We conducted this systematic review and meta-analysis to evaluate the efficacy of high-flow nasal oxygenation (HFNO) compared with that of standard oxygen therapy in adult patients undergoing procedural sedation.

**Methods:**

We identified randomized controlled trials published before November 2020 based on PubMed, Embase, and Cochrane Library databases and ClinicalTrials.gov registry. Intraprocedural desaturation [peripheral oxygen saturation (SpO_2_) < 90%] was evaluated as the primary outcome. The secondary outcomes were the lowest SpO_2_, need for airway intervention, oxygen therapy-related complications, and patient, operator, and anesthetist’s satisfaction.

**Results:**

Six trials with a total of 2633 patients were reviewed. Patients using HFNO compared with standard oxygen therapy had a significantly lower risk of intraprocedural desaturation [risk ratio 0.18, 95% confidence interval (CI) 0.04-0.87]. The lowest intraprocedural SpO_2_ in HFNO group was significantly higher than that in standard oxygen therapy group (mean difference 4.19%, 95% CI 1.74-6.65).

**Conclusions:**

Compared with standard oxygen therapy, HFNO may reduce the risk of desaturation and increase the lowest SpO_2_ in adult patients undergoing sedation for medical procedures.

## Background

Medical procedures cause anxiety, pain, and discomfort, such as gastrointestinal endoscopy, bronchoscopy, and dental treatment. These procedures are frequently performed with sedation to reduce patients’ discomfort and apprehension, contributing to a better quality of examination or intervention (Meining et al. 2007). Anesthetics and analgesics used for sedation provide hemodynamic stability by attenuating the autonomic stress response (Gerstein et al. 2016). However, sedation itself may decrease respiratory drive, cause upper airway obstruction, and thereafter hypoxia during procedures (Mason et al. 2019; Amornyotin 2013). Severe hypoxia prompts airway intervention such as mask ventilation, thus interrupting the procedure. Prolonged hypoxia may lead to cardiopulmonary distress, bradycardia, permanent neurologic damage, and even death (Shirota et al. 2020; Xiao et al. 2016; Qadeer et al. 2011; Wehrmann and Riphaus 2008). Thus, it is crucial to prevent the occurrence of hypoxia while providing an adequate depth of sedation.

In procedural sedation, patients generally receive supplemental oxygen to reduce the risk of desaturation. Nasal cannulas and simple masks are conventionally recognized as standard oxygen therapy to deliver oxygen at a maximum of 15 L/min. Currently, high-flow nasal oxygen device produces heated and humidified oxygen and enables oxygen comfortably delivered at a rate up to 70 L/min (Spoletini et al. 2015). Compared with standard oxygen therapy, high-flow nasal oxygenation (HFNO) allows for rapid carbon dioxide washout (i.e., dead space washout), maintains a constant fraction of inspired oxygen (FiO_2_), produces a positive end-expiratory pressure, reduces respiratory effort, and improves patient comfort (Helviz and Einav 2018; Lee et al. 2016). A meta-analysis recently reported that HFNO may reduce hypoxia and increase minimum O_2_ saturation during intraoperative period (Spence et al. 2020). However, it remains unclear whether HFNO is more effective in preventing occurrence of desaturation in the setting of procedural sedation compared to standard oxygen therapy. Accordingly, we conducted a systematic review and meta-analysis to compare the efficacy of oxygenation between HFNO and standard oxygen therapy in patients undergoing procedural sedation.

## Methods

### Criteria of study selection

We included randomized controlled trials (RCTs) comparing HFNO and standard oxygen care in patients undergoing sedation for diagnostic or interventional procedures in which endotracheal intubation or a supraglottic device was not necessary. In the study selection, we adopted the definition of sedation and general anesthesia by the American Society of Anesthesiologists, Committee on Quality Management and Departmental Administration 2019 (American Society of Anesthesiologists, Committee on Quality Management and Departmental Administration 2019). Specifically, sedation is a drug-induced depression of consciousness; patients are arousable and have a purposeful response to verbal, tactile, or painful stimulation (American Society of Anesthesiologists, Committee on Quality Management and Departmental Administration 2019). By contrast, patients under general anesthesia are unarousable even by painful stimulation and frequently have inadequate spontaneous ventilation, necessitating airway intervention (American Society of Anesthesiologists, Committee on Quality Management and Departmental Administration 2019). Studies were also required to clearly report the inclusion and exclusion criteria for patients, medical procedures, sedation techniques, and oxygenation strategy. Studies were excluded for the following reasons: (1) inclusion of pediatric patients, defined as younger than 18 years old, (2) inclusion of critically ill patients who had respiratory failure and required endotracheal intubation, and (3) comparison of respiratory support at the time of endotracheal extubation.

### Search strategy

We searched relevant studies published before November 2020 from the PubMed, Embase, and Cochrane Library databases using the following Medical Subject Headings: (high-flow OR high flow nasal) AND (sedation)*.* The “related articles” option in PubMed was used to broaden the search, and all abstracts, studies, and citations retrieved were reviewed. In addition, we identified other studies by using the reference sections of relevant papers and by corresponding with subject experts. Finally, unpublished studies were collected from the ClinicalTrials.gov registry (http://clinicaltrials.gov/). No language restriction was applied. The systematic review described herein has been accepted by PROSPERO, an online international prospective register of systematic reviews curated by the National Institute for Health Research (CRD42020203175).

### Data extraction

Baseline and outcome data were independently retrieved by two reviewers (H.Y.L. and J.T.C.), who extracted the following data: study designs, patient characteristics, inclusion and exclusion criteria, medical procedures, sedation techniques, oxygenation strategy, intraprocedural desaturation events, lowest O_2_ saturation, need for airway intervention, patient, operator, and anesthetist satisfaction, and oxygen therapy-related complications. Decisions recorded individually by the reviewers were compared, and disagreements were resolved by a third reviewer (K.W.T.). The authors of the studies were contacted for additional information if needed.

### Appraisal of methodological quality

Two reviewers (H.Y.L. and J.T.C.) independently assessed the methodological quality of each study by using the risk of bias method recommended by The Cochrane Collaboration (Higgins et al. 2011). The following domains were assessed: adequacy of randomization, allocation concealment, outcome assessor blinding to patient information, follow-up duration, information provided to participants regarding study withdrawal, whether intention-to-treat analysis was performed, and freedom from other biases.

### Outcomes of interest

Primary outcome was the event of desaturation (SpO_2_ < 90%) during the procedure. Secondary outcomes were (1) the lowest SpO_2_, (2) need for airway intervention, including chin lift, jaw thrust, bag mask ventilation, insertion of a nasal or oral airway, and endotracheal intubation, (3) oxygen therapy-related complications, and (4) patient, operator, and anesthetist satisfaction.

### Statistical analyses

Data were analyzed using Review Manager (version 5.3; The Cochrane Collaboration, Oxford, England, UK). Meta-analysis was performed following PRISMA guidelines (Moher et al. 2009). Mean and standard deviations (SD) were estimated from the provided median and interquartile range (IQR) (Luo et al. 2018; Shi et al. 2020). Dichotomous outcomes (desaturation event, need for airway intervention, and oxygen therapy-related complications) were presented as proportions or ratios and analyzed using risk ratios (RRs) as the summary statistic. The effect sizes of continuous outcomes were reported as the weighted mean difference (WMD) [95% confidence interval (CI)]. A pooled estimate of the RR and WMD was computed using the DerSimonian and Laird random effect models (DerSimonian and Laird 1986).

Statistical heterogeneity and the inconsistency of treatment effects across studies were evaluated using Cochrane *Q* test and *I*^2^ statistics, respectively. Statistical significance was set at *p* < 0.10 for Cochrane *Q* test. Statistical heterogeneity across studies was assessed using the *I*^2^ test, which quantifies the proportion of the total outcome variability across the studies. Moreover, subgroup analyses were performed with obese and nonobese patients and eliminated the result of Riccio and colleagues due to their study population restricted to the obese (Riccio et al. 2019).

## Results

### Trial characteristics

**Fig. 1:**
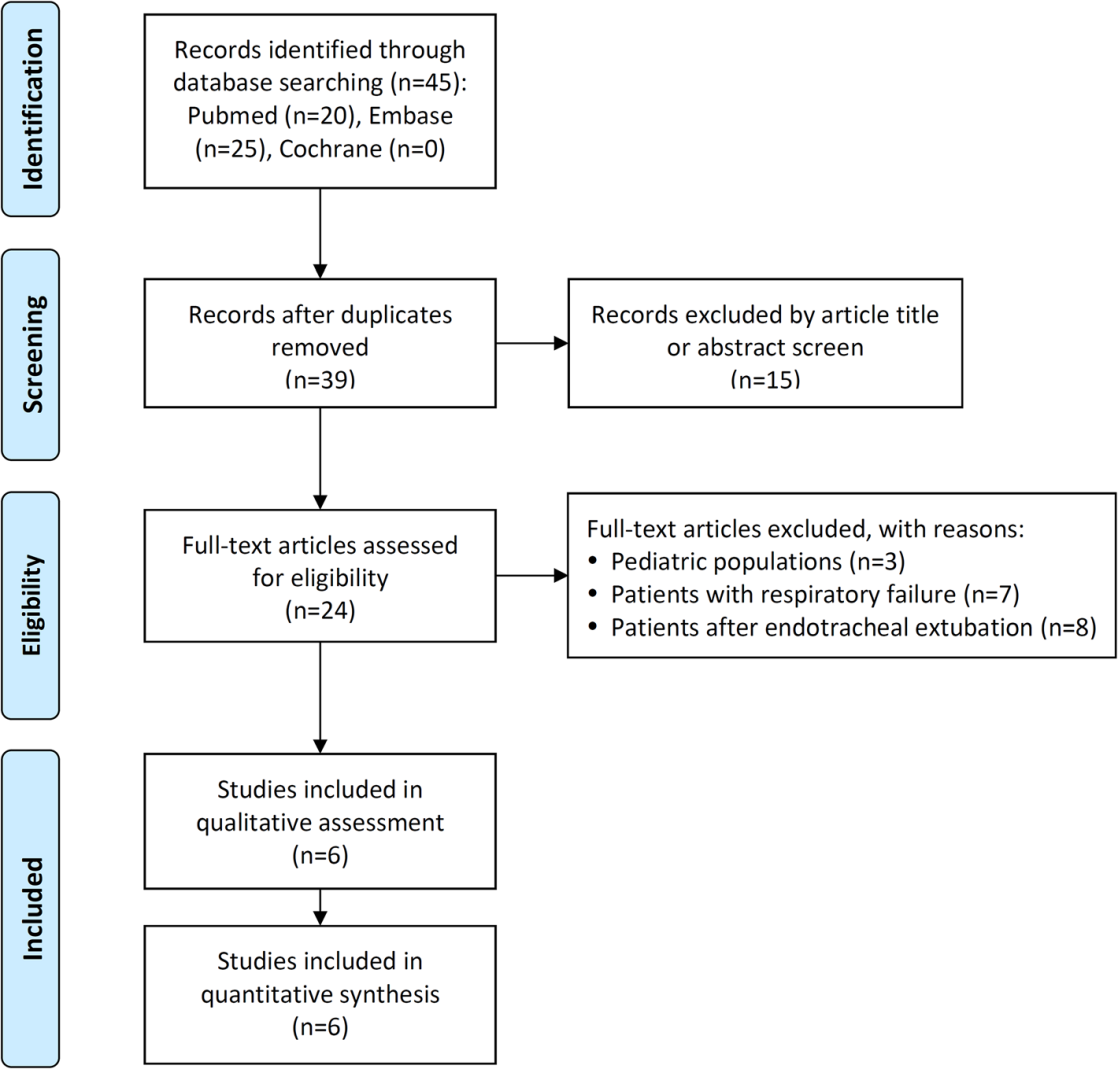
Flowchart of study selection

Figure 1 illustrates the flowchart of trial screening and selection. The initial search yielded 90 citations, of which 66 were deemed ineligible based on title and abstract screening. Next, the full texts of 24 studies were retrieved. Most of them (*n* = 18) were excluded for the following reasons: 3 included pediatric populations; 7 focused on patients with respiratory failure; 8 evaluated patients following endotracheal extubation. Six studies were finally included for analysis (Riccio et al. 2019; Ben-Menachem et al. 2020; Douglas et al. 2018; Lin et al. 2019; Teng et al. 2019; Sago et al. 2015).

These six trials were published between 2015 and 2020, with sample sizes ranging from 30 to 1994. Two of them recruited patients for bronchoscopy (Ben-Menachem et al. 2020; Douglas et al. 2018), two for upper gastrointestinal endoscopy (Lin et al. 2019; Teng et al. 2019), one for colonoscopy (Riccio et al. 2019), and one for dental treatment (Sago et al. 2015). One trial recruited patients with morbid obesity [mean body mass index (BMI) 48.5 kg/m^2^) (Riccio et al. 2019) and five recruited patients with mean BMI < 28 kg/m^2^ (Ben-Menachem et al. 2020; Douglas et al. 2018; Lin et al. 2019; Teng et al. 2019; Sago et al. 2015). For the HFNO group, all trials set the flow rate at 30–70 L/min; FiO_2_ was 100% in three trials (Douglas et al. 2018; Lin et al. 2019; Teng et al. 2019), 36 to 40% in two trials (Riccio et al. 2019; Sago et al. 2015), and not mentioned in one trial (Ben-Menachem et al. 2020). For the standard oxygen therapy group, all trials set the flow rate at 2 to 10 L/min; the oxygen was delivered through a nasal cannula in five trials (Riccio et al. 2019; Ben-Menachem et al. 2020; Lin et al. 2019; Teng et al. 2019; Sago et al. 2015) and through bite block in one (Douglas et al. 2018). Sago and colleagues divided the HFNO group into two with respect to the flow rate (30 L/min and 50 L/min) and compared them separately with the standard oxygen therapy group (Sago et al. 2015). We combined the two HFNO groups in our analysis. Two trials used propofol as the sedative agents (Riccio et al. 2019; Lin et al. 2019); one used propofol with midazolam (Sago et al. 2015); two used propofol with midazolam and alfentanil (Ben-Menachem et al. 2020; Teng et al. 2019); one used midazolam, opioids, and/or propofol at the anesthetist’s discretion (Douglas et al. 2018). Baseline patient characteristics were balanced between HFNO group and standard oxygen therapy group in all included trials (Table 1).

**Table 1:** Characteristics of the selected randomized controlled trials

Authors (year)	Inclusion criteria	No. of patients (male, %)	Age, years [mean (SD)]	BMI [mean (SD)]	Sedation technique and level of sedation	Oxygenation strategy
Riccio et al. (2019)	BMI > 40 for elective colonoscopy	H: 28 (14) S: 31 (13)	H: 54 (8) S: 59 (7)	H: 48 (7) S: 49 (10)	Induction with lidocaine up to 100 mg + propofol 30–100 mg. Maintenance with propofol 120–150 μg/kg/min ideal body weight. Keep RASS −3 to −4.	H: FiO_2_ 36–40%, up to 60 L/min S: FiO_2_ 36–40%, 4 L/min via N/C
Ben-Menachem et al. (2020)	Age ≥ 18, lung transplant recipients, for TBLB	H: 37 (60) S: 39 (74)	H: 55 (12) S: 56 (12)	H: 25 (4) S: 25 (4)	Premedication with midazolam 1–3 mg. Induction and maintenance with propofol and alfentanil.	H: 30–50 L/min S: 4–10 L/min via N/C
Douglas et al. (2018)	Age ≥ 18, for endobronchial ultrasound	H: 30 (63) S: 30 (63)	H: 63 (14) S: 63 (14)	H: 27 (6) S: 27 (6)	Induction and maintenance with midazolam, opioids, and/or propofol. Keep MOAA/S equal to 4.	H: FiO_2_ 100%, start with 30 L/min, then 30–70 L/min after sedation S: 10–15 L/min via a bite block
Lin et al. (2019)	Outpatients for elective gastroscopy	H: 994 (42) S: 1000 (41)	H: 48 (19) S: 47 (19)	H: 23 (3) S: 23 (3)	Induction and maintenance with intermittent boluses of propofol 0.5 mg/kg. Keep RSS > 4.	H: start with 2 L/min via N/C, then FiO_2_ 100%, 60 L/min after sedation S: 2 L/min via N/C
Teng et al. (2019)	Age 20-80, ASA class I or II, for outpatient EGD	H: 50 (38) M: 51 (37) S: 51 (43)	H: 47 (15) M: 51 (12) S: 52 (13)	H: 23 (4) M: 23 (4) S: 23 (4)	Induction with midazolam 0.05 mg/kg + alfentanil 0.2 μg/kg. Maintenance with TCI of propofol. Keep MOAA/S < 2.	H: FiO_2_ 100%, 30 L/min M: 5 L/min direct to nose and mouth S: 5 L/min via N/C
Sago et al. (2015)	For dental treatment under sedation	H1: 10 (NR) H2: 10 (NR) S: 10 (NR)	H1: 37 (12) H2: 39 (11) S: 40 (15)	H1: 22 (3) H2: 22 (3) S: 23 (3)	Induction with midazolam 0.05 mg/kg + TCI of propofol. Maintenance with TCI of propofol, plasma concentration 1.2–2 μg/mL. Keep bispectral index 70.	H1: FiO_2_ 40%, 30 L/min H2: FiO_2_ 40%, 50 L/min S: 5 L/min via N/C

*Abbreviations*: *ASA* American Society of Anesthesiologists, *BMI* body mass index, *EGD* esophagogastroduodenoscopy, *FiO*_*2*_ fraction of inspired oxygen, *H* high-flow nasal oxygenation, *M* mandibular advancement device, *MOAA/S* Modified Observer’s Assessment of Alertness/Sedation Scale, *N/C* nasal cannula, *NR* not reported, *RASS* Richmond Agitation-Sedation Scale, *RSS* Ramsay Sedation Scale, *S* standard oxygen therapy, *TBLB* transbronchial lung biopsy, *TCI*, target continuous infusion

Table 2 summarizes the methodological quality of the included trials. All studies had acceptable methods of randomization. Three used intention-to-treat analysis (Riccio et al. 2019; Douglas et al. 2018; Sago et al. 2015), and the other three used per-protocol analysis (Ben-Menachem et al. 2020; Lin et al. 2019; Teng et al. 2019). The proportion of patients lost to follow-up was acceptable (< 20%) in all trials. Participants were not blinded in any trial owing to the study design.

**Table 2:** Methodological quality assessment of the included trials

Authors (year)	Bias from randomization process	Deviations from intended interventions	Bias caused by missing outcome data	Bias in outcome measurement	Bias in selection of reported results	Overall risk of bias
Riccio et al. (2019)	Low risk	Some concerns	Low risk	Low risk	Low risk	Some concerns
Ben-Menachem et al. (2020)	Low risk	Some concerns	Low risk	Low risk	Low risk	Some concerns
Douglas et al. (2018)	Low risk	Some concerns	Low risk	Low risk	Low risk	Some concerns
Lin et al. (2019)	Low risk	Low risk	Low risk	Low risk	Low risk	Low risk
Teng et al. (2019)	Some concerns	Low risk	Low risk	Low risk	Low risk	Some concerns
Sago et al. (2015)	Some concerns	Some sconcerns	Low risk	Low risk	Low risk	Some concerns

### Desaturation event

**Fig. 2:**
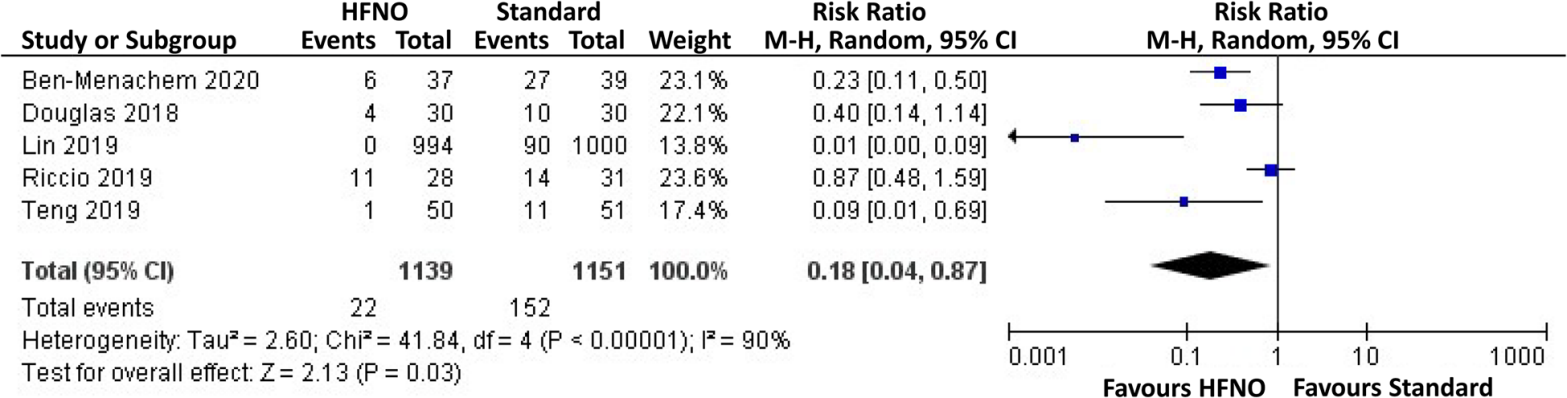
Forest plot of the comparison of desaturation event between HFNO and standard oxygen therapy groups

Five trials compared intraprocedural desaturation events between HFNO and standard oxygen therapy (Riccio et al. 2019; Ben-Menachem et al. 2020; Douglas et al. 2018; Lin et al. 2019; Teng et al. 2019). Four trials defined desaturation or hypoxic events as SpO_2_ < 90% (Riccio et al. 2019; Ben-Menachem et al. 2020; Douglas et al. 2018; Teng et al. 2019). Lin and co-workers categorized low-SpO_2_ events into subclinical respiratory depression (SpO_2_, 90 to 94%), hypoxia (SpO_2_, 75 to 89% for < 60 s), and severe hypoxia (SpO_2_, < 75% or 75 to 89% for > 60 s) (Lin et al. 2019). For this study (Lin et al. 2019), we considered hypoxic and severe hypoxic events as desaturation events in the data synthesis. HFNO was associated with a significantly lower risk of intraprocedural desaturation (RR, 0.18, 95% CI, 0.04-0.87) compared with standard oxygen therapy (Fig. 2). Subgroup analysis revealed that the reduced risk of HFNO was augmented for nonobese patients (RR, 0.11, 95% CI, 0.02-0.65).

### Lowest SpO_2_

**Fig. 3:**
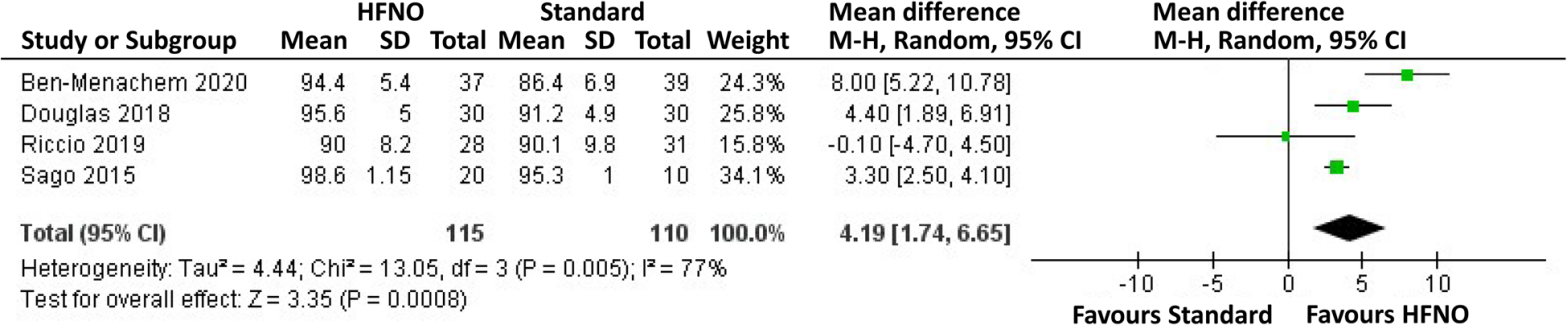
Forest plot of the comparison of lowest SpO_2_ between HFNO and standard oxygen therapy groups

Four trials compared the intraprocedural lowest SpO_2_ between HFNO and standard oxygen therapy (Riccio et al. 2019; Ben-Menachem et al. 2020; Douglas et al. 2018; Sago et al. 2015). Two reported the data as mean and SD (Riccio et al. 2019; Sago et al. 2015), and two as median and IQR (Ben-Menachem et al. 2020; Douglas et al. 2018). Therefore, mean and SD were estimated from the provided median and IQR (Luo et al. 2018; Shi et al. 2020). Sago and colleagues presented the values as a figure of mean and SD, and the statistic number was estimated from the scale on the figure (Sago et al. 2015). The intraprocedural lowest SpO_2_ of HFNO group was significantly higher than that of standard oxygen therapy group (WMD, 4.19%, 95% CI, 1.74-6.65) (Fig. 3). Subgroup analysis showed a larger difference (WMD, 4.99%, 95% CI, 2.34-7.63) between groups for nonobese patients.

### Need for airway intervention

**Fig. 4:**
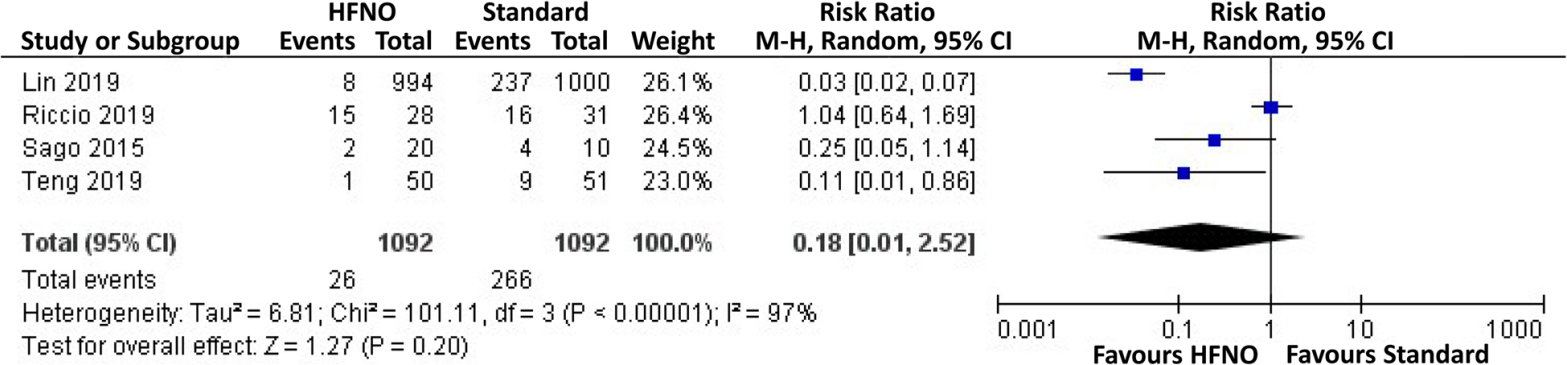
Forest plot of the comparison of need for airway intervention between HFNO and standard oxygen therapy groups

Five trials compared the incidence of airway intervention between HFNO and standard oxygen therapy (Riccio et al. 2019; Ben-Menachem et al. 2020; Lin et al. 2019; Teng et al. 2019; Sago et al. 2015). Timing of airway intervention was set at SpO_2_ < 94% or obvious airway obstruction (Ben-Menachem et al. 2020), SpO_2_ < 95%^21^, SpO_2_ < 90%^19^, SpO_2_ < 95% for > 1 min^24^, and not mentioned in two trials (Ben-Menachem et al. 2020; Teng et al. 2019). Four trials reported the number of patients who received airway intervention (Riccio et al. 2019; Lin et al. 2019; Teng et al. 2019; Sago et al. 2015), and one reported the number of interventions for each patient (Ben-Menachem et al. 2020). The result of Ben-Menachem and colleagues was not comparable and was thus excluded from our meta-analysis (Ben-Menachem et al. 2020). There was no significant difference in the risk of airway intervention between groups (RR, 0.18, 95% CI, 0.01-2.52) (Fig. 4). Subgroup analysis revealed that HFNO was linked to a lower risk of airway intervention compared to standard oxygen therapy in nonobese patients (RR, 0.09, 95% CI, 0.02-0.36).

### Complications

Three trials evaluated oxygen therapy-related complications (Douglas et al. 2018; Lin et al. 2019; Teng et al. 2019). No complication was reported in either group in one trial (Douglas et al. 2018). Lin and co-workers reported that 17 patients in the HFNO group experienced dry nasal mucosa or nasal pain and provided no description of complications in the standard oxygen therapy group (Lin et al. 2019). Teng and colleagues reported that 2 patients had nasal dryness and itching in the standard oxygen therapy group and none in the HFNO group (Teng et al. 2019). Considering the inconsistent evaluation of related complications in these trials, we did not compare oxygen therapy-related complications between groups.

### Satisfaction of patients, operators, and anesthetists

Three trials compared patient satisfaction between HFNO and standard oxygen therapy (Ben-Menachem et al. 2020; Douglas et al. 2018; Sago et al. 2015), and no significant difference was noted between groups (WMD, −0.02, 95% CI, −0.19 to 0.14).

Three trials compared operator satisfaction between HFNO and standard oxygen therapy (Ben-Menachem et al. 2020; Douglas et al. 2018; Sago et al. 2015). Satisfaction was measured using a 5-point scale ranging from 1 (very dissatisfied) to 5 (very satisfied) in two trials (Ben-Menachem et al. 2020; Sago et al. 2015). One trial used the rating in opposite directions (Douglas et al. 2018), and the results were converted into the same direction as those of the other two trials. No significant difference was observed in operator satisfaction between groups (WMD, 0.11, 95% CI, −0.25 to 0.46).

Two trials compared anesthetist satisfaction between HFNO and standard oxygen therapy (Ben-Menachem et al. 2020; Douglas et al. 2018). One trial demonstrated that HFNO had significantly better anesthetist satisfaction than standard oxygen therapy (4-5 vs. 2-4, *p* < 0.001) (Ben-Menachem et al. 2020). However, another study observed no difference between groups (*p* = 0.28) (Douglas et al. 2018). Meta-analysis showed no significant difference in anesthetist satisfaction between groups (WMD, 1.0, 95% CI, −0.76 to 2.76).

## Discussion

Our analysis showed that HFNO was associated with a reduced risk of desaturation compared to standard oxygen therapy in procedural sedation. In addition, patients using HFNO had higher minimum SpO_2_ than standard oxygen therapy. However, there was no significant difference in the risk of airway intervention, oxygen therapy-related complications, patient, operator, or anesthetist satisfaction. Our findings support that HFNO may serve as a better oxygenation technique to prevent the occurrence of hypoxia compared to conventional oxygen therapy in patients undergoing sedation for medical procedures.

Several systematic reviews have studied the effect of HFNO in the perioperative period (Spence et al. 2020; Chaudhuri et al. 2020). Spence and colleagues demonstrated that in the intraoperative period, HFNO reduces the risk of O_2_ desaturation and increases minimum O_2_ saturation and safe apnea time compared with conventional oxygenation, consistent with our results (Spence et al. 2020). In contrast, Chaudhuri and co-workers focused on the peri-intubation period and found that HFNO is not associated with severe desaturation, serious complications, apneic time, length of intensive care unit stay, or overall survival (Chaudhuri et al. 2020). The authors analyzed severe desaturation defined as SpO_2_ < 80%, which is likely to have a lower rate compared to our definition SpO_2_ < 90% (Chaudhuri et al. 2020). In addition, their analyses mixed perioperative non-hypoxemic patients and critically ill hypoxemic patients (Chaudhuri et al. 2020). Underpowered statistics and heterogeneous populations may explain the non-significant difference in desaturation risk between HFNO and standard oxygen therapy.

Obesity is associated with increased sedation-related complications, including hypoxia (Kilic et al. 2019; Jirapinyo and Thompson 2016). Most trials in our analysis recruited patients with BMI < 28 kg/m^2^, except for the study of Riccio and colleagues (Riccio et al. 2019). Our subgroup analyses indicated that the reduced desaturation risk and higher minimum SpO_2_ associated with HFNO were augmented in nonobese patients. Similarly, the intergroup difference in need for airway intervention was only significant in nonobese patients. Conversely, a recent clinical trial demonstrated that HFNO achieved a longer safe apnea time and higher minimum SpO_2_ compared to facemask oxygenation in patients with morbid obesity undergoing anesthesia induction (Wong et al. 2019). More studies are required to determine whether HFNO is effective in preventing desaturation in obese patients undergoing procedural sedation.

The depth of sedation may have an influence on respiratory-related complications (American Society of Anesthesiologists, Committee on Quality Management and Departmental Administration 2019). Deep sedation may exert a higher risk of hypoventilation, airway obstruction, and desaturation, particularly when propofol is used as the primary sedative (Sheahan and Mathews 2014). Our selected studies used different assessment tools for depth of sedation. Two trials maintained the patients at moderate sedation with Modified Observer’s Assessment of Alertness/Sedation Scale (MOAA/S) equal to 4 and Ramsay Sedation Scale > 4 (Douglas et al. 2018; Lin et al. 2019); one trial maintained the patients at moderate-to-deep sedation with Richmond Agitation-Sedation Scale of −3 to −4 (Riccio et al. 2019); two trials maintained deep sedation with bispectral index around 70 and MOAA/S < 2 (Teng et al. 2019; Sago et al. 2015). However, the available data were insufficient for subgroup analysis. Further research should evaluate the effect of varying depths of sedation on the effectiveness of HFNO in oxygenation and prevention of desaturation.

The use of higher FiO_2_ itself may reduce risk of desaturation in procedural sedation. A nasal cannula with an oxygen flow rate of 4 to 10 L/min provides FiO_2_ 30 to 35%, and oxygen through bite block with a flow rate of 10 L/min provides FiO_2_ approximately 35% (Ting et al. 2012). In our analysis, only two trials used the same FiO_2_ in both groups (Riccio et al. 2019; Sago et al. 2015), and three trials used FiO_2_ 100% in the HFNO group (Douglas et al. 2018; Lin et al. 2019; Teng et al. 2019). The difference in applied FiO_2_ between groups may confound the effect of oxygenation techniques on desaturation risk. Nevertheless, HFNO may improve oxygen delivery by minimizing oxygen dilution and reducing dead space compared to conventional oxygen therapy regardless of FiO_2_ (Lee et al. 2016).

Our study covered the procedures requiring sedation, including gastrointestinal endoscopy, bronchoscopy, and dental treatments. Although these procedures are typically performed outside the operating room, patients with the potential to convert to conventional surgery or general anesthesia may undergo these procedures in the operating room (Youn et al. 2015). In addition, operations undertaken with a varying level of sedation are also common in the operating room. The oxygenation strategy to prevent desaturation is of equal importance to these procedures and operations, especially in the field of airway management.

Our study has several limitations. First, some trials had a small sample size of 10 per treatment group (Sago et al. 2015). Second, it is difficult to conduct a meta-analysis for some outcomes due to insufficient data, such as oxygen therapy-related complications. Third, the satisfaction score was subjective and was likely to be biased as the participants were not blinded to assigned intervention. Fourth, the cost of HFNO is much higher than conventional oxygen therapy, but we did not analyze cost effectiveness (Eaton Turner and Jenks 2018). Fifth, there is considerable heterogeneity in the type and dosage of sedative agents and patients’ baseline clinical conditions among the included trials. Finally, pediatric, hypoxemic, and extubated patients were excluded from the analysis. Therefore, our results are not applicable to these populations.

## Conclusions

Our systematic review and meta-analysis demonstrated that HFNO may reduce the risk of desaturation and increase the lowest SpO_2_ in patients receiving sedation for medical procedures compared to standard oxygen therapy. HFNO can be considered as the choice of oxygen therapy in procedural sedation. Future studies should focus on high-risk patients, such as those with respiratory distress and morbid obesity.

## Data Availability

The materials are retrieved from published data.

## Abbreviations

BMI: Body mass index
CI: Confidence interval
FiO_2_: Fraction of inspired oxygen
HFNO: High-flow nasal oxygenation
IQR: Interquartile range
MOAA/S: Modified Observer’s Assessment of Alertness/Sedation Scale
RCTs: Randomized controlled trials
SD: Standard deviation
SpO_2_: Peripheral oxygen saturation
WMD: Weighted mean difference
